# Effect of *Pochonia chlamydosporia* Endophytism and *Meloidogyne incognita* Parasitism on WRKYs and Defense Gene Expression in Tomato Roots

**DOI:** 10.3390/plants12061284

**Published:** 2023-03-11

**Authors:** Laura C. Rosso, Francesca Romano, Isabella Pentimone, Aurelio Ciancio, Mariantonietta Colagiero

**Affiliations:** 1 Consiglio Nazionale delle Ricerche, Istituto per la Protezione Sostenibile delle Piante, CNR, Via G. Amendola 122/D, 70126 Bari, Italy; 2 Medical Genetics, Department of Precision Medicine, Università degli Studi della Campania, via Luigi De Crecchio 7, Naples 80131, Naples, Italy

**Keywords:** defense response genes, root-knot nematode, tomato, transcription factor

## Abstract

The expression of WRKY transcription factors and plant defense-related genes was studied in the roots of Apulian tomato (*Solanum lycopersicum*) cv Regina di Fasano (accessions MRT and PLZ) endophytically colonized by *Pochonia chlamydosporia* and parasitized or not by the root-knot nematode (RKN) *Meloidogyne incognita*. The effect on plant growth, nematode parasitism and histological aspect of the interaction were considered. The association of *P. chlamydosporia* with RKN-parasitized MRT plants increased the total biomass and shoot fresh weight in comparison with healthy plants and with those only parasitized by RKN, without the endophyte. However, the PLZ accession showed no significant difference in the observed biometric parameters. The number of RKN-induced galls per plant was not affected by endophytism eight days after inoculation. No histological changes were observed in the nematode feeding sites in the presence of the fungus. Gene expression analysis showed an accession-specific response to *P. chlamydosporia* with differential activation of WRKY-related genes. No significant change was found for *WRKY76* expression in nematode-parasitized plants compared with control roots, confirming cultivar susceptibility. Data indicate genotype-specific responses of the WRKY genes to parasitism examined in roots with nematodes and/or endophytic *P. chlamydosporia*. At 25 days post-inoculation with *P. chlamydosporia*, no significant difference was observed in the expression of defense-related genes in both accessions, suggesting that salicylic acid (SA) (*PAL* and *PR1*) and jasmonate (JA) related genes (*Pin II*) are not active during endophytism.

## 1. Introduction

The use of antagonistic microorganisms to manage plant pathogens and pests is considered beneficial due to a major interest in environmental protection issues and the development of resistance to pesticides in target organisms.

In many plant and pathogen or pest interactions, experimental data have shown that some antagonistic species stimulate the expression of genes increasing plant resistance by sustaining a defense response, strengthening the innate antimicrobial arsenals [1,2,3,4]. The first step in achieving effective biocontrol is to identify and understand the mechanisms that take place in the interactions between the organisms directly implicated. It has been observed, for example, that the colonization of the root system by some rhizobacteria can lead to the activation of defense systems both in the roots and in the aerial parts far from the colonized region [1]. The same mechanism has also been described for some fungi, such as mycorrhizae or other endophytes [2,3,4]. Molecular biology techniques allow detailed studies on many of these interactions, including those occurring between plants and microorganisms, analyzing pathways, levels of gene expression or repression, and the factors involved.

This study focused on the fungus *Pochonia chlamydosporia*, a hyphomycete that elicits interest as a bio-pesticide and growth promoter, exploiting a wide range of trophic niches. This fungus is a parasite of nematode and mollusk eggs and a hyperparasite of some fungi [5,6]. It can also survive as a saprotroph in soil in the absence of nematodes and/or host plants. In the rhizosphere, *P. chlamydosporia* colonizes roots, in which it has been reported as endophytic on a range of mono- and dicot species [7,8,9]. Inoculum of *P. chlamydosporia* promoted plant development in the early stages of growth and development, increasing stem and root weight in barley, tomato and wheat [9]. Molecular studies have provided evidence that *P. chlamydosporia* is capable of inducing plant defense phenotypes, characterized by anatomical changes and production of different metabolites [7]. Barley and tomato plants respond to fungus colonization with the formation of papillae and other cell wall appositions. Callose, which in many plant interactions is related to defense, is found in papillae colonized by the hyphae of *P. chlamydosporia*. Cell wall modifications induced by the fungus in roots does not impede root colonization, suggesting that *P. chlamydosporia* plays an indirect role in defense response modulation [10]. Phenolic compounds, including lignin, which are active in plant defense, have been reported in the papillae of root cells colonized by the fungus [11]. Both callose and lignin have a structural role, enhancing the tissues’ mechanical resistance to penetration, with antimicrobial properties.

At the molecular level, modifications of the tomato transcriptome by endophytic *P. chlamydosporia* highlight a specific modulation of stress-responsive transcripts related to selective activation of defense pathways, likely induced by the fungus when establishing a persistent endophytic lifestyle [12]. The differential ability of *P. chlamydosporia* isolates to induce systemic resistance against *M. incognita* has also been reported in tomato in split-root experiments [13,14].

When *Pochonia* spp. colonize roots, they act as true endophytes without inducing any damage. However, the capacity to colonize and proliferate in roots varies greatly among isolates [13]. Gene expression studies have suggested specific and time-dependent relationships linking host plants and *P. chlamydosporia* in the presence of biotic stress factors, functional to a systemic, but complex, activation of defense genes [15]. The efficiency of the root defense system depends on a complex network of molecules, including the activation not only of genes from defense response pathways (hormones and secondary metabolite synthesis) but also of regulatory elements, such as WRKY transcription factors. These proteins are involved in plant growth regulation and development. However, their main functional role is the regulation of the plant’s response to biotic and abiotic stress factors [16,17]. WRKYs involved in regulating parasitism by plant parasitic nematodes have been studied in Arabidopsis, soybean, rice and tomato hosts. Grunewald et al. [18] showed that feeding site establishment by the cyst nematode *Heterodera schachtii* in Arabidopsis was associated with an increased expression of the auxin-inducible gene *AtWRKY23*. Ali et al. [19] demonstrated that among 59 WRKY genes present on the Arabidopsis ATH1GeneChip array, 28 were downregulated and 6 were upregulated. Overexpression of the respective genes demonstrated that their downregulation was important for nematode development, likely via interference with plant defense signals.

Yang et al. [20] characterized the soybean WRKY gene family and identified five WRKY genes (*GmWRKY154*, *GmWRKY62*, *GmWRKY36*, *GmWRKY28* and *GmWRKY5*) conferring high resistance to the soybean cyst nematode (>70% reduction in the number of cysts). Strong upregulation was detected for transcripts encoding OsWRKY62, OsWRKY59 and OsWRKY13 following rice infection by the root-knot nematode (RKN) *Meloidogyne graminicola* [21,22]. In tomato, a genome-wide computational analysis revealed 81 WRKY genes [23]. At least 19 of them were differentially expressed in response to *M. javanica* [24,25], with four WRKYs implicated in the tomato–RKN interaction identified. The WRKYs functioning in the pathway mediated by RKN resistance genes showed that paralogues—i.e., *SlWRKY72a* and *b*—were upregulated during the pest resistance (incompatible) reaction mediated by *Mi-1*. Gene silencing of these two genes in tomato resulted in a clear reduction in *Mi-1*-mediated resistance against RKN [26]. Subsequently, Atamian et al. [27] showed that silencing *SlWRKY70* attenuated *Mi-1*-mediated resistance against RKN and that the transcript levels of *SlWRKY70* were upregulated by SA and suppressed by methyl jasmonate (MeJA).

The aim of this work was to obtain gene expression information to identify molecular factors that contribute to improving the resistance of tomato to biotic stress, as well as increasing current knowledge on the mechanism that takes place during the double biological interaction with the endophyte. We analyzed the expression of WRKY transcription factors and defense response-related genes in the roots of two Apulian tomato accessions (*cv* Regina di Fasano), endophitically colonized by *P. chlamydosporia* and successively parasitized by *M. incognita*.

## 2. Results

### 2.1. Effect on Plant Growth

The growth performance of MRT and PLZ accessions differed. When measured at the beginning of the assay, PLZ seedlings showed a better performance compared to MRT plants. Such superior performance was maintained until the end of the assay (48-day-old plants), with PLZ consistently showing higher biometric values than those of MRT (Table 1). The interaction with *P. chlamydosporia* (treatment F) increased in MRT the total biomass and shoot fresh weight in comparison with healthy plants (C, control) and with plants also inoculated with *M. incognita* (treatment FN). In addition, the root fresh weight was higher in roots with *P. chlamydosporia* endophitism (treatment F) when compared to roots under double interaction (treatment FN) (Table 1). No significant difference in biometric parameters was observed among PLZ plants, suggesting the occurrence of a tolerant response to nematode parasitism. The level of *M. incognita* parasitism, evaluated as the number of galls per plant eight days after inoculation, was not affected by endophytism in either accession.

**Figure 1:**
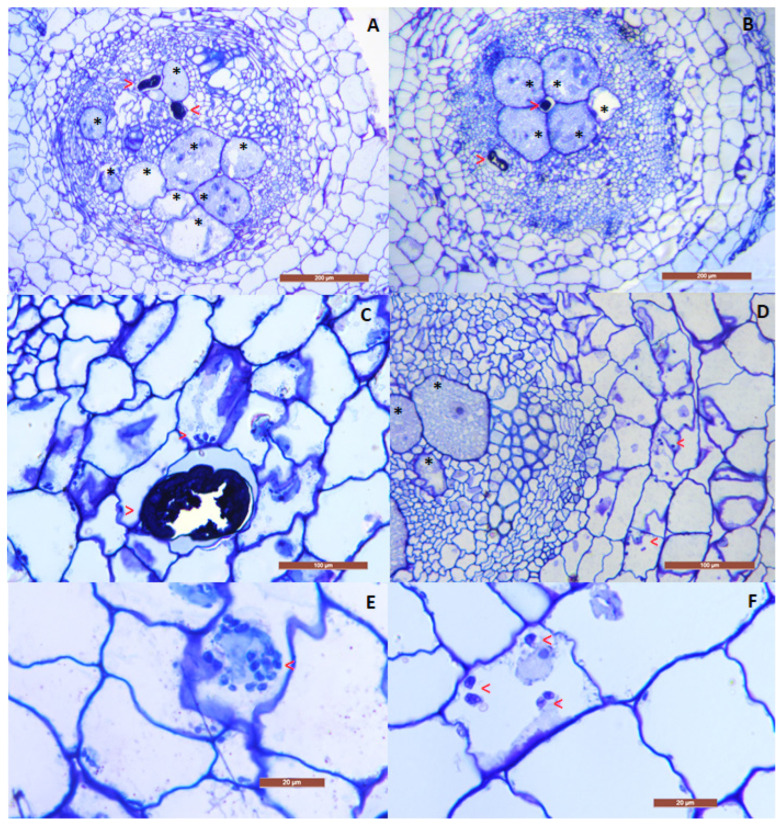
Sections from galls induced by *M. incognita* in the roots of tomato accessions MRT (**A**) and PLZ (**B**), showing alternated xylematic and phloematic arcs and feeding sites. Chlamydospores and hyphae of *P. chlamydosporia* occurred in the cortical area of both MRT (**C**,**E**) and PLZ (**D**,**F**) roots. Asterisks show giant cells, and arrows indicate nematodes and chlamydospores. Scale bars: (**A**,**B**) = 200 µm; (**C**,**D**) = 100 µm; (**E**,**F**) = 20 µm.

Successful endophytic colonization by *P. chlamydosporia* was verified for both accessions, which showed the presence of conidia and chlamydospores in the cortical areas of roots (Figure 1C–F). Sections showed vascular bundles alternating endodermal xylematic and phloematic arcs in both accessions. Changes in the central cylinder anatomy were observed due to the presence of the nematode feeding sites, with remarkable hyperplasia. The parenchymal cells and vessels were pushed outward and, in some cases, completely compressed. No difference was found in the nematode feeding sites in the presence of the fungus. The nematode managed to penetrate the host plant, reaching the central cylinder and inducing the formation of giant cells (Figure 1A,B). Cortical parenchyma around nematodes did not show necrotic areas and/or damaged cells. Each gall contained 3–4 females in both treatments (N and FN) and accessions.

Data suggest that, at early stages, endophytism did not affect the rate of nematode infestation, as no significant difference was observed in the number of galls in treated plants at 8 dpi for both accessions (Table 1).

The endophyte managed to reach the root cortical area, where it could be found at 24 dpi, without reaching the central cylinder, consistent with previous reports [8]. Root colonization, estimated by qPCR, showed that *P. chlamydosporia* colonized both healthy and parasitized roots of both accessions, and no significant difference was observed in fungal DNA amounts between treatments F and FN.

The standard curve for qPCR obtained by representing the cycle thresholds (Ct) vs. the log of 10-fold serial dilution of DNA appeared accurate and reproducible (R^2^ = 0.992). The estimated amounts of fungal DNA in the MRT (F and FN roots) were 5.1 × 10^−9^ ± 4.9 × 10^−9^ and 1.1 × 10^−6^ ± 4.2 × 10^−7^ ng, respectively. In PLZ, F and FN roots yielded 6.9 × 10^−9^ ± 3.7 × 10^−9^ and 1.9 × 10^−6^ ± 7.3 × 10^−7^ ng, respectively. *P. chlamydosporia* DNA was not detected in the control plants.

### 2.2. WRKY Gene Expression

The primers used in molecular analysis to amplify the tomato genes coding for WRKY 25, WRKY 44, WRKY 76, PAL 5, PR 1, and PIN II yielded PCR products with the expected sizes and single dissociation curves.

**Figure 2:**
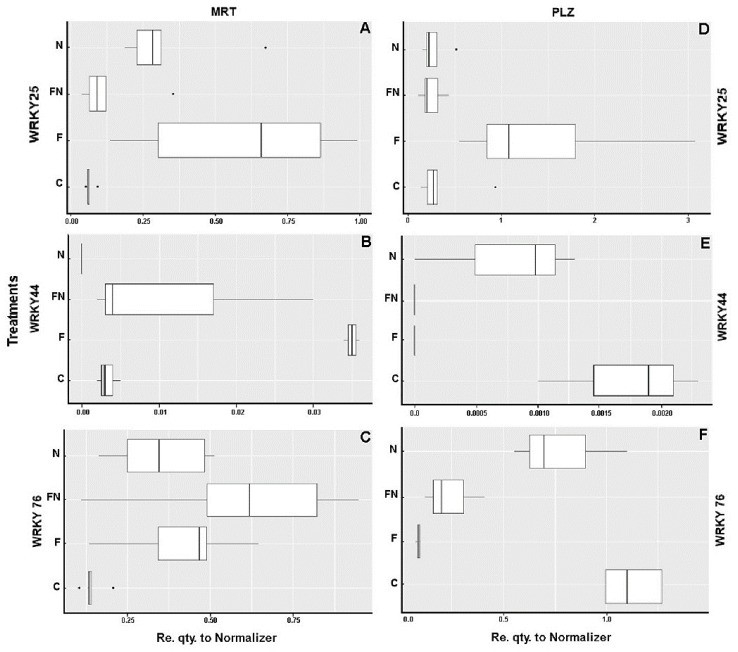
Expression of *WRKY 25*, *44* and *76* genes in tomato accessions MRT (**A**–**C**) and PLZ (**D**–**F**). Each boxplot displays the first and third quartiles (lower and upper hinges), the median (vertical bar in the box), and the largest and smallest value (upper and lower whiskers), with outlying individual points (see Table 2 for description of treatments). Gene expression levels are shown along the x-axes as relative quantities of transcripts compared to the actin normalizer gene.

Accession MRT showed, in association with *P. chlamydosporia*, transcription levels for *WRKYs 25*, *44* and *76* significantly higher than those of the control (Table 2, Figure 2A–C). *WRKY 25* showed the highest expression in the presence of the endophyte alone (F), whereas the double interaction (fungus + nematode, FN) reduced its expression compared with each association alone (F and N). Major *WRKY 44* expression was observed in treatment F. The double interaction (FN) reduced its expression, whereas in roots associated only with the nematode (N), its transcripts were undetectable. When *M. incognita* was also present, *WRKY 76* showed higher expression values in FN treatment compared to C and N. For the inoculated plant (F), a significant increase in *WRKY 76* expression was also observed, compared with control roots (C) (Table 2, Figure 2A–C).

Accession PLZ showed activation of the selected WRKY genes that was partially different from MRT. The highest upregulation level of *WRKY 25* was found in the PLZ roots inoculated with *P. chlamydosporia*, compared with the other conditions (C, FN and N), which showed similar expression levels (Table 2, Figure 2D). No significant difference from MRT was also observed for *WRKY 25* in the presence of *M. incognita*, although at 8 dpi, a lower number of transcripts was found. The amount of *WRKY 44* transcripts in PLZ was around 10-fold lower compared with MRT accession. Its expression was highest in the control and reduced in the double interaction with the endophyte (F and FN). Similarly, F and FN roots showed lower expression of *WRKY 76* compared with either the C or N treatments (Figure 2D and Table 2).

### 2.3. Defense Gene Expression

**Figure 3:**
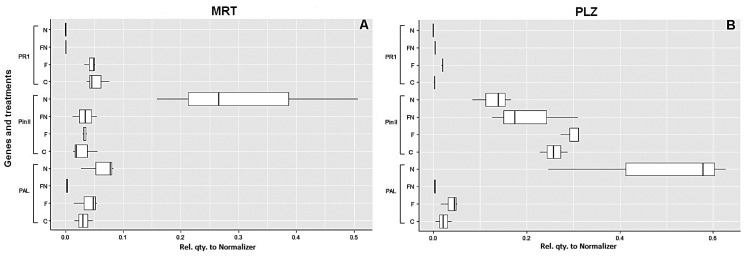
Expression of defense response genes *PR1*, *Pin II* and *PAL 5* by treatments in tomato cv Regina di Fasano accessions MRT (**A**) and PLZ (**B**) (see Table 2 for description of treatments). Gene expression levels are shown along the x-axes as relative quantities of transcripts compared to the actin normalizer.

The expression of defense genes differed between the two tomato genotypes, as well as among some of the conditions tested (Table 3, Figure 3). In MRT, inoculation with *M. incognita* downregulated *PR1* in the treatments with nematodes, with or without *P. chlamydosporia*. This gene was expressed at higher levels in the control plants or in those inoculated with the fungus alone. In this accession, transcript coding for PIN II showed the highest differential upregulation in the plants inoculated with the nematode only (treatment N, Figure 3A). For this gene, the plants inoculated with the endophyte (F and FN) did not show any significant difference from the control roots. For *PAL 5*, the highest expression was observed in plants with only *M. incognita* (N). However, the gene was also expressed at similar levels in treatments F and C and was significantly downregulated when the plants were also inoculated with the endophyte, indicating a possible inhibition of *PAL 5* in the double interaction (FN).

Accession PLZ (Table 3, Figure 3B) differed from MRT, as PR1 was also downregulated in the control plants, with a higher (although of low intensity) expression in plants inoculated with the endophyte alone (Figure 3B). As in MRT, PR1 also appeared inhibited in PLZ by *M. incognita*, as shown by downregulation in treatments FN and N (Table 3, Figure 3A,B). Differing from MRT, PLZ showed upregulation of *PIN II* in plants inoculated with *P. chlamydosporia* (F), at a level significantly higher when compared with the nematode inoculated plants (F vs. N). PLZ data indicated that the nematode and the endophyte had opposite effects on the expression of this gene. While the fungus alone induced *PIN II* expression at higher levels than the control, *M. incognita* inhibited or reduced its transcription, as shown by intermediate values observed in FN, with the lowest expression in N. The expression of *PAL 5* in PLZ showed trends similar to MRT, with the highest expression in roots with *M. incognita* (N) and the lowest in the double interaction (FN).

## 3. Discussion

New and recent updates of the tomato genome annotation, fundamental for an accurate gene functional definition, yielded some incongruencies for a number of sequences also annotated and reported in previous studies. In some cases, the sequence annotations changed between subsequent genome versions, as studies preceding the last genome update reported sequence annotations that no longer corresponded to the new ones. Further analyses of published sequences have been needed, both to keep useful genetic information coherent across genome versions and to avoid incongruent interpretations.

The lower expression, during the FN double interaction, of *WRKY25* in accession MRT (computationally annotated as WRKY transcription factor 23 in ITAG release 2.4, with a 63% similarity of positive strand with AtWRKY22) suggests a possible repression of the negative regulator in RKN-parasitized plants, increasing resistance to RKN. Studies conducted on *Arabidopsis thaliana* plants associated with the cyst nematode *Heterodera schactii* showed an upregulation of *AtWRKY23* in the first 12 h after attack, suggesting a mechanism of direct activation of *WRKY23* transcription by the nematode. The upregulation of *WRKY23* in the early stages of infection seems to be necessary for parasite establishment [18]. In *Populus* spp., the suppression of *Ptwrky23* de-regulated genes related to the redox state, decreasing the accumulation of lignin. This effect could interfere with the defense response, explaining the increase in susceptibility [8,28].

Homologous *AtWRKY22* in *Arabidopsis thaliana* has a central role in induced systemic resistance (ISR) triggered against *V. dahliae* by the plant growth-promoting rhizobacterium *Paenibacillus alvei*. *AtWRKY22*-mutant lost the protection mediated by the PGPR [29]. The expression of *WRKY25* in inoculated tomato roots could result in a negative regulation of defense responses, suggesting that the endophytism of *P. chlamydosporia* alone involves a modulation of the plant defense responses. In this condition, the expression of *WRKY25* may favor fungus establishment in the roots. However, the plant defense response rapidly resumed after nematode penetration and establishment, as shown by the low expression of *WRKY25* in treatments N and FN (Figure 3A,D).

*WRKY44* responses during the interaction with *P. chlamydosporia* confirmed the presence of different regulatory mechanisms between the two accessions. While the MRT roots activated *WRKY44* in the double interaction, PLZ inhibited its transcription. Additionally, the two accessions showed opposite responses in the presence of nematodes or *P. chlamydosporia* alone (Figure 3B,E). Studies conducted on *WRKY44* in *Gossypium hirsutum* indicated that it positively regulates pathogen-induced plant disease resistance, as shown in transgenic *Nicotiana benthamiana* vs. *Ralstonia solanacearum* [30]. Signaling molecules (SA, MeJA and ET) induce the expression of *GhWRKY44*, suggesting that it could be involved in the SA or JA/ET signaling pathway. Studies carried out with *GhWRKY44*-overexpressing plants under *R. solanacearum* infection indicated that its overexpression could enhance resistance by inhibiting the accumulation of pathogen-induced ROS. The differences in gene expression levels observed in our study indicate tomato germplasm-specific responses to *P. chlamydosporia* in which the activation of *WRKY44* likely reacts to different ROS induced by *M. incognita* or the endophyte.

An equal expression of *WRKY76* (computationally annotated as WRKY transcription factor 72 in ITAG release 2.4) was observed in parasitized, as well as endophytically colonized, MRT plants compared to the control. In this case, the reactions of the two accessions differed, as PLZ showed responses to treatments different from MRT (Figure 3C,F). Previous studies reported an upregulation of *WRKY72* after *M. incognita* inoculation in resistant tomato cv Motelle but not in susceptible cultivars [26]. The functional relationship between the *Mi-1* gene (conferring *M. incognita* resistance) and *WRKY72* seems not to be bound to the de-repression of basal defense responses but rather to a direct defense activation mechanism or an efficient suppression of nematode effector induction [31]. It is interesting to observe that *WRKY76* expression in MRT synergically increased in *P. chlamydosporia*-colonized plants in the presence of *M. incognita*. The activation of WRKY76 suggests a specific response activated by the fungus during the biological double interaction, which could be associated with a resistance response. Conversely, repression was observed for this gene in the fungus-inoculated PLZ plants, which also showed a constitutive expression level of *WRKY76* in the control.

Upregulation of *PIN II* in parasitized roots was previously reported for RKN-resistant plants [31]. Upregulation has been associated with the activation of the octanoid pathway, a mechanism active in the plant resistance response [31,32]. Upregulation of *PIN II* mostly occurred in RKN-parasitized MRT roots, which could indicate a more resistant response. Conversely, its inhibition in PLZ could be indicative of a more susceptible condition.

As expected, *PAL 5* was mostly expressed in the presence of RKN in both accessions. Its inhibition in the double interaction (FN) is consistent with observations by Zhang et al. [33], who also reported inhibition of *PAL* in plants inoculated with *P. chlamydosporia* PC-170, 15 and 30 days after nematode inoculation. Downregulation of *PAL* occurred in roots with *P. chlamydosporia* and *M. incognita*, compared with RKN-only infested plants, whereas the fungus alone did not show significant differences from the control (Figure 3). PAL is the first enzyme of phenylpropanoid metabolism, a key element in the SA pathway, involved in abiotic and biotic stress responses. Studies of PAL enzymatic activity, carried out after a 7 day interaction in tomato with *P. chlamydosporia* and *M. javanica*, showed that its activity was detrimental for the colonization of the fungus and the induction of resistance [32]. A study conducted on wheat demonstrated a strong induction of *PAL* (AevPAL1) by the cereal cyst nematode [33]. These observations suggest that root colonization by *P. chlamydosporia* could be affected by the defense responses related to SA in the presence of a biotrophic pathogen, such as *M. incognita*. Our observations also indicate a tomato genotype-dependent response to this enzyme.

Downregulation of *PR1* in both accessions in the presence of RKN confirms the inverse relationship between local expression of *PR1* and nematode susceptibility. Similar results were also reported for *NPR1* expression in plants inoculated with *P. chlamydosporia* PC-170 at 30 days after nematode inoculation [33]. As previously reported in the association of *A. thaliana* with *H. schachtii* [34] and also for the cv Regina di Fasano, the success of parasitism may likely depend on local suppression of the SA pathway. No difference in the expression of *PR1* was observed between roots with *P. chlamydosporia* and RKN in both accessions. Instead, *P. chlamydosporia*-colonized roots of MRT showed a higher level of upregulation of PR1, similar to the control.

The profiles of gene expression observed represent a single time frame of relationships likely lasting and changing in a more prolonged time period, as reported in the interaction between tomato and *Trichoderma harzianum*, where the increase in expression of plant defense genes occurred not earlier than 5 weeks after inoculation with *M. incognita* [35]. Although at 8 dpi, a direct link between expression levels of WRKY and defense genes could not be determined, data suggest similarities in the expression profiles among treatments between *WRKY25* and *PR1* for both accessions. Similar trends in expression profiles were also observed between *WRKY25* and *PIN II* for the PLZ accession only and *PAL* for both accessions, except nematode-parasitized PLZ plants.

## 4. Materials and Methods

### 4.1. Plant, Nematode and Fungus Source

Two local accessions of *Solanum lycopersicum* cv Regina di Fasano, MRT and PLZ, collected from farms in the province of Brindisi (Italy), were used. Tomato seeds were washed (0.1% Tween 20), surface sterilized in 2.5% Na hypochlorite, and germinated on 1.5% water agar at 25 °C in the dark. Subsequently, the seedlings were transferred to 10 cm diameter pots containing a soil and sand mix (50:50, *v*:*v*) that was previously sterilized. Each pot received 100 mL of sterile water followed by incubation at 26 °C in a growth chamber (Sanyo MLR-351) with a photoperiod of 16:8 h of light:dark. Inoculation was carried out with isolate DSM 26985 of *P. chlamydosporia*, present in the collection at the Institute for Sustainable Plant Protection (IPSP) of the CNR and deposited at DSMZ (Leibniz Institute, Braunschweig, Germany). The plants (23 days old) were inoculated around the roots in the assays with *P. chlamydosporia*, adding 7 mL of a water solution containing 10^5^ conidia and other fungus propagules mL^−1^.

A population of *M. incognita*, MILEV-L4, present in the collection at IPSP, was reproduced and maintained on the susceptible tomato variety UC82. The hatched J2s were collected after 10 days of incubation of infected roots in tap water at 25 °C, under continuous air ventilation from a peristaltic pump. The tomato seedlings were inoculated with 1 mL of larval suspension (about 300 larvae/plant) 17 days after fungus inoculation.

### 4.2. Tomato-Pochonia Chlamydosporia-RKN Assay

The experimental design included tomato inoculated with DSM 26985 (F), DSM 26985 and MILEV-L4 (FN), or inoculated only with MILEV-L4 (N). An uninoculated plant was used as control (C). Each treatment consisted of 4 replications, for a total of 16 plants for both accessions. After 48 days from germination (8 days after *M. incognita* inoculation), the plants were explanted, and biometric parameters were recorded. A sample of around 100–120 mg of root tissue and galls was taken for each sample and stored at −80 °C.

### 4.3. RNA Extraction and Retrotranscription

TRIzol reagent was used for RNA extraction following the manufacturer’s instructions. The root tissue was disrupted by immersion in liquid nitrogen and pulverized using a pestle and mortar. RNA was purified by adding 1 mL of TRIzol and 0.2 mL of chloroform by incubation for 2–3 min at room temperature. After cold centrifugation (4 °C) at 12,000× *g* for 15 min, the aqueous layer was removed, and RNA was precipitated by the addition of 0.5 mL of 100% isopropanol and centrifugation at 12,000× *g* for 10 min at 4 °C. After washing with 75% ethanol, the RNA pellet was dissolved in nuclease-free pure water. RNA integrity was checked by electrophoresis on 1% (p/v) agarose gel, and the concentration was determined using Nanodrop™ spectrophotometer (Thermo Fisher Sc., Waltham, MA, USA). The retrotranscription reaction was performed in a total volume of 20 µL containing an initial amount of 500 ng of RNA. cDNA synthesis was produced using the QuantiTect^®^ Reverse Transcription Kit (Qiagen, Hilden, Germany), following the manufacturer’s instructions. Elimination of DNA contamination was included as the first step of the retrotranscription kit.

### 4.4. Primer Design

The defense-related genes encoding phenylalanine ammonia lyase (*PAL*), pathogenesis-related protein 1 (*PR1*) and proteinase inhibitor II (*PIN II*) were examined as marker genes involved in the SA and JA pathways. Primer sequences and genome locations are reported in Tolba et al. [15].

Specific WRKY primers were designed from available sequences of *S. lycopersicum* SL4.0 (https://solgenomics.net/organism/Solanum_lycopersicum/genome/ (accessed on 1 October 2021) (Table 4). The primers were selected after confirmation of protein conserved domains by BLASTX (https://blast.ncbi.nlm.nih.gov/), using Primer3 and the primer BLAST tool (https://www.ncbi.nlm.nih.gov/tools/primer-blast/ (accessed on 1 October 2021)). Forward and reverse WRKY 76 primers were designed on locus Solyc05g007110 WRKY transcription factor 76 (*SlWRKY76*), computationally annotated as WRKY transcription factor 72 and previously indicated as WRKY72 in the Tomato genome version (ITAG release 2.4). Locus Solyc10g011910 for WRKY transcription factor 25 (*SlWRKY25*) was used for forward and reverse primers, computationally annotated as WRKY transcription factor 23, indicated as WRKY23 in the previous Tomato genome version (ITAG release 2.4), with a 63% similarity (positive) with AtWRKY22. The sequence of WRKY transcription factor 44 from locus Solyc10g084380 was selected for forward and reverse primers (Table 4).

### 4.5. Real-Time PCR and Gene Expression Analyses

Real-time amplification reactions were performed using a reaction mixture containing SYBR Green (Applied Biosystem, Waltham, MA, USA) in the presence of 500 nM of each primer and 1 μL of cDNA (about 25 ng), in a final volume of 15 μL. The reactions were conducted in a Mx3000P™ thermocycler (Stratagene, San Diego, CA, USA), with the following thermal profile: a first cycle at 94 °C for 4 min, followed by 40 cycles at 94 °C for 30 s, annealing temperature for 30 s, and 72 °C for 20 s. For each reaction, a dissociation curve was produced at the end-point reaction to confirm amplicon specificity. The threshold cycle (Ct) values were determined for two biological assays with three technical replicates each. The amplification efficiencies (E) were calculated with the Miner program [36] using the fluorescence values recorded during amplification. The initial relative amount of the transcripts was normalized based on the amplification of the actin constitutive gene using primers reported in [15] (Table 4). Quantitative data were automatically calculated from the MXpro QPCR Software (Stratagene) based on the relative quantification method, DCT [37,38], which uses the Ct values to estimate the amount of a target gene, compared to a normalizing gene (Normalized expression = (1 + E target) ^−∆Ct target^/(1 + E actin) ^−∆Ct actin^). For comparative analysis, the data obtained were submitted to the Least Significant Difference (LSD) Fisher test (*p* ≤ 0.05).

### 4.6. Histopathology

Histopathological analysis was set up to evaluate the effect of *P. chlamydosporia* on nematode parasitism, in particular the effect on the nematode feeding site in the presence and absence of the fungus. Galls and healthy portions of roots of the FN- and N-treated plants were hand-dissected and fixed in a mix of 1.5% glutaraldehyde and 3% paraformaldehyde in 10 mM phosphate-buffered saline (PBS) containing 150 mM NaCl (pH 7.2) for 3 h at room temperature, dehydrated in a graded ethanol series to absolute ethanol and then embedded in LR White acrylic resin (Sigma, St. Louis, MO, USA), left to polymerize overnight at 60 °C. Around 15–20 root samples were taken for each treatment, which were cut by an ultramicrotome to obtain 2.5 μm thick sections. After cutting, the sections were collected on a slide, stained with 1% Toluidine blue solution (1 g Na tetrahydroborate, 1 g of Toluidine blue, 100 mL H_2_O) and observed under a light microscope.

### 4.7. Quantitation of P. chlamydosporia Endophytic Colonization

Endophytic colonization of *P. chlamydosporia* was verified by qPCR. The roots were collected and stored at −80 °C. Total DNA was extracted from around 100 mg of tissue using a plant/fungi DNA isolation kit (Norgen Biotek, Thorold, ON, Canada) following the manufacturer’s instructions. Total DNA was quantified by a Nanodrop™ spectrophotometer (Thermo Fisher Sc., MA, USA), and a 50 ng/µL concentration solution was prepared for PCR reactions. For *P. chlamydosporia* detection and quantification, qPCR experiments were conducted using an Aria PCR device (Agilent Scientific Instruments, Santa Clara, CA, USA). The alkaline serine protease (VCP1) was selected as the target gene using primers For0 and Rev0 [15]. Amplification reactions were performed in a 15 µL volume with 2× SYBR green master mix (AllGene, Madison, WI, USA), 50 ng of total DNA and 500 nM of primer. The thermal profile was 95 °C for 3 min, 40 cycles at 95 °C for 30 s, 52 °C for 20 s, and 72 °C for 10 s. A standard curve was constructed using serial dilutions from 1 ng to 1 pg of genomic DNA of the *P. chlamydosporia* DSM 26985. The initial amount of fungal DNA contained in total root DNA was calculated by the correlation of quantification cycle (Cq) values with Cq values in the standard curve. Data significance was evaluated by applying Student’s *t*-test (*p* ≤ 0.05).

## 5. Conclusions

The beneficial effects observed in the growth of MRT in association with *P. chlamydosporia* appear not to be bound to the induction of defense responses. Roots of Regina di Fasano tomato at 25 days post inoculation with *P. chlamydosporia* did not show significant differences in the expression of defense-related genes in both accessions, suggesting that genes for SA-related pathways (*PAL* and *PR1*) and JA (*PINII*) are not active during endophytism. Our results suggest that during the early phases of parasitism by *Meloidogyne* and the concomitant effect of fungal endophytic colonization, a genotype-specific response of WRKY genes could be involved. WRKY genes showed different expression profiles between the two accessions. Although more data are required to assess direct links of the WRKY genes with the three defense genes examined, a similar expression profile in response to treatments could be observed only between *WRKY25* and *PR1* in accession PLZ, with a partial correspondence in MRT but limited to treatments including *P. chlamydosporia*. Plant interaction with *P. chlamydosporia* stimulates the expression of genes that counteract the negative effects of the RKN. The mechanism of action and further applications in agriculture remain to be explored, but the possibility of modulating gene expression through biocompatible tools encourages future efforts.

## Figures and Tables

**Table 1:** Biometric parameters and infection data of the two tomato accessions (MRT and PLZ) tested 15 days post *P. chlamydosporia* inoculation (eight days after *M. incognita* inoculation). Treatments, N = inoculated with *M. incognita* MILEV-L4; FN = inoculated with *P. chlamydosporia* DSM 26985 and MILEV-L4; F = inoculated with DSM 26985 and C = un-inoculated, control plants. Data are the mean ± standard deviation (SD) of four biological replicates.

Acc.	Total Fresh Weight (g)	Shoot Fresh Weight (g)	Root Fresh Weight (g)	Stem Length (cm)	N. of Root Galls	N. Galls/g Root
MRT C	13.8 ± 3.7	8.1 ± 3.0	5.7 ± 1.7	12.1 ± 1.4		
MRT F	22.5 ± 5.2 *	16.4 ± 3.8 *	6.1 ± 1.6	15.6 ± 3.0		
MRT FN	13.8 ± 1.4 **	11.3 ± 1.4 **	2.5 ± 0.6 **	12.3 ± 2.1	59.0 ± 21.5	25.8 ± 13.0
MRT N	19.1 ± 5.4	13.7 ± 2.6 *	5.4 ± 3.6	11.5 ± 0.8 **	62.6 ± 36.9	17.2 ± 12.9
PLZ C	24.0 ± 3.9	16.8 ± 2.7	7.1 ± 1.8	15.0 ± 1.0		
PLZ F	22.7 ± 7.7	16.4 ± 5.5	6.2 ± 2.7	14.1 ± 1.1		
PLZ FN	22.6 ± 5.0	15.9 ± 2.3	6.7 ± 2.7	13.3 ± 0.7	85.0 ± 29.5	14.5 ± 8.1
PLZ N	19.0 ± 6.9	14.1 ± 4.3	4.9 ± 3.0	15.7 ± 4.3	62.2 ± 17.6	17.9 ± 14.9

Symbols * and ** show significant differences (LSD, Fisher, *p* ≤ 0.05) vs. control (C) and *P. chlamydosporia* inoculated (F) plants, respectively.

**Table 2:** Double entry table reporting significant up- (bold) or downregulated WRKY genes between the different treatments (N = inoculated with *M. incognita* MILEV-L4; FN = inoculated with *P. chlamydosporia* DSM 26985 and MILEV-L4; F = inoculated with DSM 26985 and C = un-inoculated, control plants) for the two tomato accessions (MRT and PLZ). LSD Fisher test, *p* ≤ 0.05, ns = not significant.

Accessions		MRT			PLZ	
Treatments	F	FN	N	F	FN	N
**C**	*WRKY **25***, ***44***, ***76***	*WRKY **76***	ns	*WRKY **25**,76*	*WRKY 76*, *44*	ns
**F**		*WRKY 25*, *44*	ns		*WRKY 25*	*WRKY 25*, ***76***
**FN**			*WRKY 76*			*WRKY **76***

**Table 3:** Double entry table reporting significant gene expression response among the different treatments and accessions (upregulated genes are shown in bold; see Table 2 for description of treatments; LSD, Fisher test, *p* ≤ 0.05; ns = not significant).

Accessions		MRT			PLZ	
Treatments	F	FN	N	F	FN	N
**C**	ns	PR1	**PIN II**, PR1	ns	ns	PIN II, **PAL**, PR1
**F**		PR1	**PIN II**, PR1		ns	PIN II
**FN**			**PIN II**, **PAL**			**PAL**, PR1

**Table 4:** Primer description used for qPCR amplification. *S. lycopersicum* sequences available in *S. lycopersicum* genome version 4.0 (https://solgenomics.net/organism/Solanum_ lycopersicum/genome (accessed 1 October 2021)). *P. chlamydosporia* sequence available in GenBank (https://www.ncbi.nlm.nih.gov/nuccore/AJ427460 (accessed 1 October 2021)).

Organism	Name	Locus/Accession Number	Nucleotide Sequence	
*S. lycopersicum*	WRKY 76	Solyc05g007110	Forward	5′-AAGATTTCCACTTATTTTTCCACTGAG-3′
			Reverse	5′-GATAAAGAACCAAGCAGGCGT-3
	WRKY25	Solyc10g011910	Forward	5′-TGTTTCCGATGTTGGGTTGG-3
			Reverse	5′-GTAGAGCAATCAACGACCGC-3′
	WRKY44	Solyc10g084380	Forward	5′-AGATTCTGCTGAAGGAAGTAA-3′
			Reverse	5′-AATGTACTTTCCCCTGATGTA-3′
	ACTIN	Solyc03g078400	Forward	5′-AGGCAGGATTTGCTGGTGATGATGCT-3′
			Reverse	5′-TACGCATCCTTCTGTCCCATTCCGA-3′
*P. chlamydosporia*	VCP1	AJ427460	For0	5′-CTCGAGGCTGCCCAAC-3′
			Rev0	5′-TGCATGCACTAGGCTCGG-3′
